# Alanylglutamine Relieved Asthma Symptoms by Regulating Gut Microbiota and the Derived Metabolites in Mice

**DOI:** 10.1155/2020/7101407

**Published:** 2020-12-29

**Authors:** Shao-Kun Liu, Li-Bing Ma, Yu Yuan, Xiao-Ying Ji, Wen-Jin Sun, Jia-Xi Duan, Qing-Ping Zeng, Binaya Wasti, Bing Xiao, Jian-Fei Zheng, Ping Chen, Xu-Dong Xiang

**Affiliations:** ^1^Pulmonary and Critical Care Medicine, The Second Xiangya Hospital, Central South University, Changsha, Hunan 410011, China; ^2^Research Unit of Respiratory Disease, Central South University, Changsha, Hunan 410011, China; ^3^Diagnosis and Treatment Center of Respiratory Disease, Central South University, Changsha, Hunan 410011, China; ^4^Department of Respiratory Medicine, The Affiliated Hospital of Guilin Medical University, Guilin 541001, China; ^5^Institute of Respiratory Diseases, Guilin Medical University, Guilin 541001, China; ^6^Department of Respiratory Medicine, The Seventh Affiliated Hospital, Sun Yat-sen University, Shenzhen 518106, China

## Abstract

**Objective:**

Allergic asthma is a chronic inflammatory disease, which seriously affects the life quality of patients, especially children. Alanylglutamine is a nutritional supplement with potential protective and anti-inflammatory effects, but its function in allergic asthma remains elusive. In this study, we focused on the investigations of the roles and functional mechanism of Alanylglutamine in asthma.

**Methods:**

Ovalbumin (OVA) induction was utilized to establish a mouse asthma model. 16S rDNA sequencing was performed to compare the diversity of intestinal microorganisms under different treatments. Gas chromatography was utilized to screen the intestinal microbe-short-chain fatty acids in the stool. The lung tissue was extracted to determine signaling pathways, including AMPK, NF-*κ*B, mTOR, STAT3, IKK*β*, TGF-*β*, and IL-1*β* through Western blot or RT-qPCR.

**Results:**

It was observed that Alanylglutamine reduced the cytokine in OVA-induced allergic asthma mice. H&E staining showed obvious pneumonia symptoms in the asthma group, while Alanylglutamine alleviated the inflammatory infiltration. Alanylglutamine reversed gut microbiota compositions in OVA-induced allergic asthma mice and enhanced the butyric acid level. The protective role of Alanylglutamine may be associated with the gut microbiota-butyric acid-GPR43 pathway in asthma mice. In contrast to the OVA group, Alanylglutamine activated the protein expression of P-AMPK/AMPK and inhibited the protein expression of P-mTOR/mTOR, P-P65/P65, P-STAT3/STAT3, P-IKK*β*/IKK*β*, TGF-*β*, and IL-1*β*, with similar effects from butyric acid.

**Conclusion:**

The results indicated that Alanylglutamine might be beneficial for asthma, and its effect was achieved through the regulation on microbiota and the derived metabolites. The therapeutic effects might be associated with AMPK, NF-*κ*B, mTOR, and STAT3 signaling pathways. These findings will help identify effective therapeutic direction to alleviate allergic inflammation of the lungs and airways.

## 1. Introduction

Asthma, also known as bronchial asthma, is a respiratory disease with complex etiology, involving a variety of cells and different molecular mechanisms in the lungs and airways [1]. It is a chronic inflammatory disease, which seriously affects the life quality of patients, especially children [2]. According to the latest scientific statistics, more than 300 million people suffer from asthma worldwide [3, 4]. The total incidence of asthma for children under 5 years old is 23/1,000 per year, and 4.4/1,000 for adolescents aged from 12 to 17 [3, 5]. Corticosteroids inhibit inflammation in asthmatic airways, which can be used in asthma therapy currently [6]. Although it is effective in alleviating acute symptoms, its adverse reactions, such as cardiotoxicity, are causing increasing concern during long time therapy. Besides, there are growing numbers of patients who are recognized as corticosteroid-resistant [7]. Therefore, it is urgent to develop effective and safe drugs to relieve asthma symptoms.

Alanylglutamine is a nutritional supplement consisting of the amino acids of L-glutamine and L-alanine. It is a stable water-soluble dipeptide with potential protection and absorption promoting activity. According to previous studies, Alanylglutamine plays an important role in the inflammatory injury of lung tissue. For example, Alanylglutamine can improve acute lung injury induced by endotoxin (LPS) through regulating Th17/Treg [8]. It also relieved intestinal epithelial cells with LPS-induced inflammation and barrier function damage [9]. It was confirmed that oral administration of both free amino acid (alanyl and glutamine) and dipeptide form (Alanylglutamine) can significantly alleviate LPS-induced inflammation [10]. As allergic asthma is an inflammation-related disease, we are interested in whether Alanylglutamine could exert a protection role in the asthma symptoms.

In recent years, accumulative evidence suggested that the intestinal functions are closely related with lung mucosal immune organs. Intestinal-pulmonary axis regulation plays an important role in respiratory diseases. Intestinal microorganisms may affect lung and respiratory diseases, such as lung infection, asthma, and chronic obstructive pulmonary disease [11]. Studies have reported that flora transplantation into the intestinal tract could change the microecology of the intestine, thereby affecting the immune and metabolic functions with therapeutic effects [12]. In the preliminary experiment, we transplanted the flora of Alanylglutamine on ordinary mice into sterile mice and established an ovalbumin (OVA) model to observe the phenotype. It was found that Alanylglutamine relieved asthma symptoms through intestinal flora. Further researches are necessary to identify their inner associations regarding the combined therapeutic effects for the asthma.

In this study, we intend to verify the relationship among Alanylglutamine dipeptide, gut microbiota, and derived metabolites in the OVA asthma model. We will examine the allergic inflammation of the lungs and airways under different treatments from the perspective of improving intestinal microorganisms by Alanylglutamine. In addition, we will investigate the expressions of related molecules, and discuss the possibility of enhancing the treatment of asthma with related drugs.

## 2. Material and Methods

### 2.1. Animal and Asthmatic Model

The experiment animals utilized in this research were maintained following the protocol approved by the Institutional Animal Care and Use Committee of Second Xiangya Hospital. Adult male BALB/c mice of 8 weeks old, weighing about 20 grams, were all purchased from Well-bio (Changsha, China) and categorized into three groups: the control group, the OVA model, and the Alanylglutamine treatment+OVA model (Alanylglutamine) (*n* = 10). All mice were placed in specific pathogen conditions and maintained at a 12 h light-dark cycle, with diet freedom. In the OVA model group, 20 *μ*g OVA (Sigma-Aldrich, USA) injection was performed on days 1 and 8. According to previous research [13, 14], aerosol (with 1% OVA in PBS) stimulated for 20 minutes on day 24, day 25, and day 26 using an ultrasonic nebulizer (Omron, Vernon) to induce allergic asthmatic mice model. Mice in the Alanylglutamine group received OVA with a diet containing 0.15% Alanylglutamine. Samples were collected after 48 hours following the last treatment.

### 2.2. Antibiotic Treatment

30 adult male BALB/c mice were treated with antibiotics in drinking water for two weeks. The antibiotics contain 1 mg/mL streptomycin sulfate, 1 mg/mL gentamicin, 1 mg/mL penicillin, and 0.5 mg/mL vancomycin. Then, water was replaced and mice were further sorted into three groups: the control group, the OVA model, and the Alanylglutamine treatment+OVA model (Alanylglutamine) (*n* = 10). OVA induction and Alanylglutamine supplementation were the same with the above treatments.

### 2.3. Sodium Butyrate (NaB) Treatment

40 adult male BALB/c mice were assigned into 4 groups: the OVA model, the OVA+NaB, the OVA+antibiotics, and the OVA+antibiotics+NaB (*n* = 10). OVA induction and antibiotic treatment were the same with the above treatments. NaB was ingested through gavage, with a dosage of 200 mg/kg/day.

### 2.4. Leukocyte Count in Bronchoalveolar Lavage Fluid (BALF)

After separating the lungs from the mice, who were sacrificed by intraperitoneal injection of pentobarbital sodium (150 mg/kg), 0.9 mL cold PBS with 2 mM EDTA and 2% fetal bovine serum (FBS) were instilled into them. Then, the BAL fluid was collected through a procedure according to the previous study [15]. BALF was acquired after lavage and centrifuged at 2000 g at 4°C for 5 minutes. The sediment was resuspended in 50 *μ*L PBS, and the number of cells was calculated using a hemocytometer. The collected BALF was centrifuged at 800 g, and its supernatant was utilized for analysis of the cytokine level.

### 2.5. ELISA Measurement of Cytokines

Concentrations of total leukocyte count, IFN-*γ*, IL-1*β*, IL-6, TNF-*α*, and TGF-*β*1 in BALF were measured by murine cytokine-specific Quantikine ELISA kits (eBioscience), in accordance with the manufacturers' instructions.

### 2.6. Western Blot

Tissue lysates were made in a radioimmunoprecipitation assay (RIPA) buffer containing 25 mM Tris-HCl (pH 7.2), 0.15 M NaCl, 0.1% SDS, 1% Triton X-100, 1% sodium deoxycholate, and 1 mM EDTA. Determination of protein concentration was carried out by bicinchoninic acid protein assay kit (Pierce). After being subjected to sodium dodecyl sulfate polyacrylamide gel electrophoresis, the protein was transferred to a nitrocellulose membrane. Total protein or phosphorylation was detected using a goat polyclonal antibody against rabbit or mouse after blocking. The protein bands were quantified using a digital imaging system (UVtec).

### 2.7. RT-qPCR

TRIzol® reagents (Invitrogen Life Technologies; Thermo Fisher Scientific, Waltham, MA, USA) were employed to extract total RNA. Subsequently, a PrimeScript™ RT reagent kit (Thermo Fisher Scientific) was utilized for reverse transcription. We conducted real-time RNA quantification on an ABI StepOne Plus Detection System (Applied Biosystems) using a Power SYBR Green PCR Master Mix (Applied Biosystems). The primer sequences were designed in the laboratory and synthesized by Sangon Biotech Co., Ltd. (Shanghai, China). The primers were designed as follows: AMPK forward, 5′-CGGGGTCATTCTCTATGCTT-3′, and reverse, 5′-TTTAAACCACTCGTGTTCCCT-3′; mTOR forward, 5′-ACCAACTATACCCGCTCCC-3′, and reverse, 5′-TAGTTGCCATCCAGACCCGTA-3′; P65 forward, 5′-TAGCCAGCGAATCCAGACCAACA-3′, and reverse, 5′-TGGGTCCCGCACTGTCACCT-3′; STAT3 forward, 5′-CAATACCATTGACCTGCCGAT-3′, and reverse, 5′-GAGCGACTCAAACTGCCCT-3′; and *β*-actin forward, 5′-ACATCCGTAAAGACCTCTATGCC-3′, and reverse, 5′-TACTCCTGCTTGCTGATCCAC-3′.

### 2.8. 16S rDNA Sequencing

The bacterial 16S rDNA gene of the stool was analyzed via a TIANamp Stool DNA Extraction Kit (TIANGEN Biotechnology, China) with Ribonuclease A (QIAGEN, Germany). We measured the purity and concentrations of the DNA through NanoDrop 1000 (Thermo Fisher Scientific). The primer sequences were used as follows: 341F primer: 5′-CCTAYGGGRBGCASCAG-3′, and 806R primer: 5′-GGACTACHVGGGTWTCTAAT-3′. Then, we constructed an amplicon sequencing library and performed sequencing using Illumina HiSeq 2500 (Illumina, San Diego, CA, USA). The data filtering, detection, and detachment of chimeric sequences were fulfilled by the Quantitative Insights Into Microbial Ecology (QIIME) pipeline (2019.07) and UCHIME algorithm, respectively. Sequences with similarities greater than 97% were defined as the same operational taxonomic unit (OTU) through UPARSE. Mothur and SSU data sets of the SILVA rRNA Database were applied for species annotation with a confidence threshold of 0.8. The analysis of sequencing data on the alpha and beta diversities was fulfilled by QIIME and R. According to the Kyoto Encyclopedia of Genes and Genomes (KEGG) gene function spectrum data, the overall metabolic function of the flora was converted and calculated, which was presented as a differentiated KEGG pathway analysis.

### 2.9. Determination of Short-Chain Fatty Acids (SCFAs) in Stool

The SCFAs in stool samples of the mice, including acetic acid, propionic acid, butyric acid, and its isomer isobutyric acid, were detected using Gas Chromatograph Mass Spectrometer-QP2010 (GC-MS) (Shimadzu, Tokyo, Japan). Every stool sample was homogenized with 50 mg/mL methanol by vortex for 10 seconds and treated by ultrasound for 10 minutes. Next, the mixed samples in each group were centrifuged at room temperature at 14,000 rpm for 5 minutes. The supernatant was diluted with methanol 10 times. Upon injection, 1 *μ*L of sample was evaporated at 230°C. The compounds were separated via an Agilent J&W fused silica capillary column DB-FFAP (Agilent, Santa Clara, CA, USA). After being ionized by electron impact at -70 eV at 200°C, the samples were analyzed via a quadrupole mass spectrometer. Each SCFA was identified using GCMSsolution software (Shimadzu, Japan). The concentrations of SCFAs were quantified according the peak areas of the total ion current.

### 2.10. Immunofluorescence Analysis

To determine the expression of GPR43 in eosinophils of lung tissue, we performed immunofluorescence staining on slides of paraffin-embedded lung tissue. The slides were incubated with primary GPR43 antibody overnight. Subsequently, the slides were cultured by Alexa Fluor 488 and Alexa Fluor 594 secondary antibodies (Invitrogen, Carlsbad, CA, USA) for 1 hour. Then, the slides were mounted by Vectashield (Vector Laboratories, USA) with DAPI. LSM 510 confocal microscope (Zeiss, Germany) was used for cell photography and counting.

### 2.11. H&E Staining of Lung

After being fixed with 4% paraformaldehyde and embedded in paraffin, 4 *μ*m sections of mouse lung tissues were stained with Hematoxylin and Eosin (H&E) (Beyotime, China) for standard histopathological examination. Each pathological section was observed under an optical microscope. The representative images for pathological analysis were utilized to evaluate the infiltration of inflammatory cells in the mouse airway and perivascular and alveolar cells.

### 2.12. Statistical Analysis

The data from the experiments were expressed as mean values ± standard deviation (SD). Statistical significance from different groups of mice was calculated by one-way ANOVA (>2 groups). *P* value less than 0.05 was considered to be statistically significant.

## 3. Results

### 3.1. Alanylglutamine Reduced the Cytokine Productions and Alleviated Inflammatory Infiltration in OVA-Induced Allergic Asthma Mice

**Figure 1:**
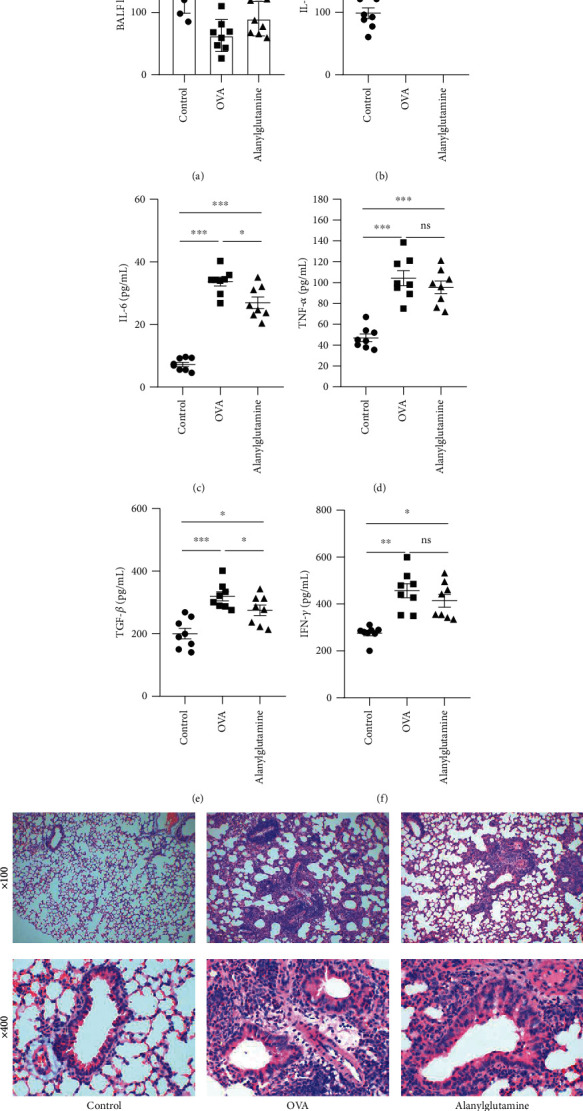
Alanylglutamine reduced the cytokine productions and alleviated inflammatory infiltration in OVA-induced allergic asthma mice: (a) BALF leukocyte count; (b) IL-1*β*; (c) IL-6; (d) TNF-*α*; (e) TGF-*β*; (f) IFN-*γ* level in BALF; (g) lung tissue morphology depicted by H&E staining. *n* = 8. ^∗^*P* < 0.05; ^∗∗^*P* < 0.01; ^∗∗∗^*P* < 0.001; ns: not significant.

In the allergic asthma model, the leukocyte count in BALF was significantly reduced (Figure 1(a)), while Alanylglutamine treatment reversed the BALF leukocyte concentration. It indicates the protective effect of Alanylglutamine in OVA-induced allergic asthma mice. As inflammation is widely associated with asthma symptoms, we measured the inflammatory indicators in BALF, including IL-1*β* (Figure 1(b)), IL-6 (Figure 1(c)), TNF-*α* (Figure 1(d)), TGF-*β* (Figure 1(e)), and IFN-*γ* (Figure 1(f)). In the OVA-treated mice, a significant inflammatory response was observed by the increased IL-1*β*, IL-6, TNF-*α*, TGF-*β*, and IFN-*γ* concentrations (*P* < 0.01). Alanylglutamine showed an anti-inflammatory effect by significantly reducing IL-1*β*, IL-6, and TGF-*β* level in BALF. Meanwhile, H&E staining showed obvious pneumonia symptoms in the OVA group, while Alanylglutamine alleviated the inflammatory infiltration (Figure 1(g)).

### 3.2. Alanylglutamine Reversed Gut Microbiota Compositions in OVA-Induced Allergic Asthma Mice

**Figure 2:**
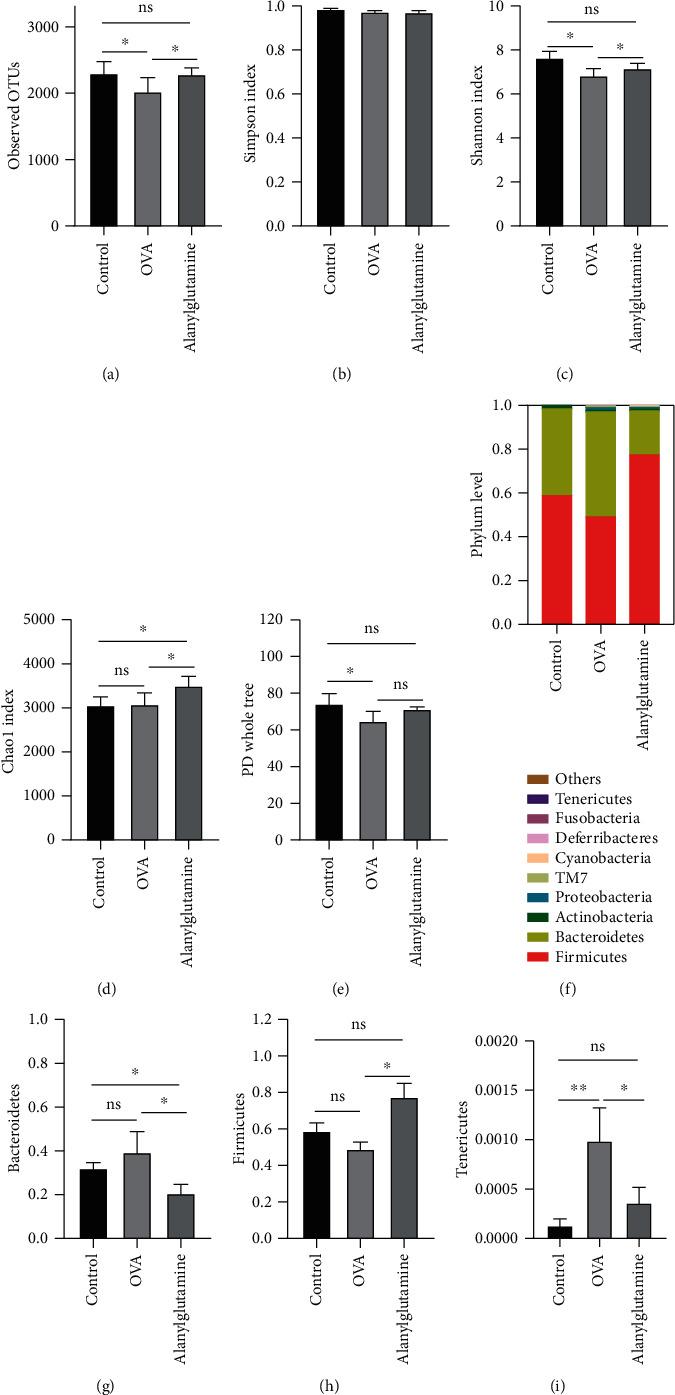
Differences of *α*-diversity index and species composition of the phylum level: (a) observed OTUs; (b) Simpson index; (c) Shannon index; (d) Chao1 index; (e) PD whole tree; (f) phylum level; (g) Bacteroidetes; (h) Firmicutes; (i) Tenericutes. OTUs: operational taxonomic units. ^∗^*P* < 0.05; ns: not significant.

Fecal microbiota diversity and compositions were further tested by 16S rDNA sequencing. To evaluate the microbiota *α*-diversity, observed OTUs and Simpson, Shannon, Chao, and PD indexes were analyzed. The results showed that OVA treatment markedly reduced bacterial *α*-diversity, with the decreased levels of observed OTUs, Shannon index, and PD, while Alanylglutamine treatment reversed the observed OTUs and Shannon index. Meanwhile, Chao1 index was increased in the Alanylglutamine group compared with the control and OVA groups (Figures 2(a)–2(e)). Fecal dysbiosis was observed in OVA-induced allergic asthma mice, which was reversed in the Alanylglutamine group.

Microbiota at the phylum was further analyzed (Figure 2(f)). It revealed that the major phyla were Bacteroidetes and Firmicutes, accounting for 95%. We found that three phyla were markedly changed in this study (Figures 2(g)–2(i)). Although OVA treatment failed to affect Bacteroidetes and Firmicutes abundance, Alanylglutamine markedly reduced Bacteroidetes and increased Firmicutes abundance, compared with the OVA group (*P* < 0.05). Meanwhile, the relative abundance of Tenericutes was significantly enhanced in OVA-challenged mice, which was reversed by dietary Alanylglutamine (*P* < 0.05). Microbiota at the genus level (top 20) was also analyzed. 11 genera were markedly altered in response to OVA or Alanylglutamine treatment (Supplementary Figure 1). Corynebacterium, Odoribacter, Staphylococcus, and Turicibacter were markedly enhanced and Parabacteroides, Streptococcus, Coprococcus, Bacteroidetes, Allobaculum, and Sutterella were decreased in OVA-challenged mice, in contrast to the control mice (*P* < 0.05). However, the relative abundance of Corynebacterium, Parabacteroides, Odoribacter, Coprococcus, Bacteroidetes, and Allobaculum was significantly reversed in Alanylglutamine-fed mice (*P* < 0.05). It showed that Alanylglutamine might improve asthma by regulating Tenericutes, Corynebacterium, Parabacteroides, etc.

PICRUSt was used to analyze functional profiling of microbial communities, including amino acid metabolism, carbohydrate metabolism, cell motility, cellular processes and signaling, energy metabolism, enzyme families, folding, sorting and degradation, genetic information processing, glycan biosynthesis and metabolism, lipid metabolism, membrane transport, metabolism, metabolism of cofactors and vitamins, nucleotide metabolism, poorly characterized, replication and repair, and transcription and translation.

**Figure 3:**
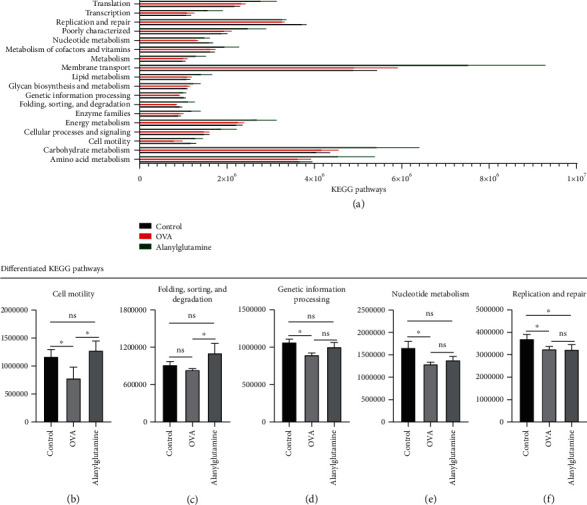
Difference analysis among samples from control, OVA, and Alanylglutamine mice: (a) KEGG pathway; (b–f) differentiated KEGG pathways for the analysis in cell mobility, folding, sorting and degradation, genetic information processing, nucleotide metabolism, and replication and repair. The vertical axis is absolute abundance of microbiota, and there was no unit for this. ^∗^*P* < 0.05; ns: not significant.

Based on the function prediction and analysis results of the KEGG pathway database, the total number of genes annotated to the pathway database in all samples was counted, and a bar chart was drawn. As shown in Figure 3(a), the gene quantity distribution of the KEGG pathway of level 1 or 2 was visually displayed. The abscissa showed the number of genes enriched in the signal pathway of three groups of samples. The ordinate showed the significantly enriched signal pathways. According to Figures 3(b)–3(f), OVA treatment markedly reduced cell motility, genetic information processing, nucleotide metabolism, and replication and repair (*P* < 0.05), while Alanylglutamine significantly increased cell motility (*P* < 0.05). Meanwhile, it also revealed that Alanylglutamine enhanced folding, sorting, and degradation.

### 3.3. Effects of Alanylglutamine on Bacterial Metabolites in the Stool in OVA-Induced Allergic Asthma Mice

**Figure 4:**
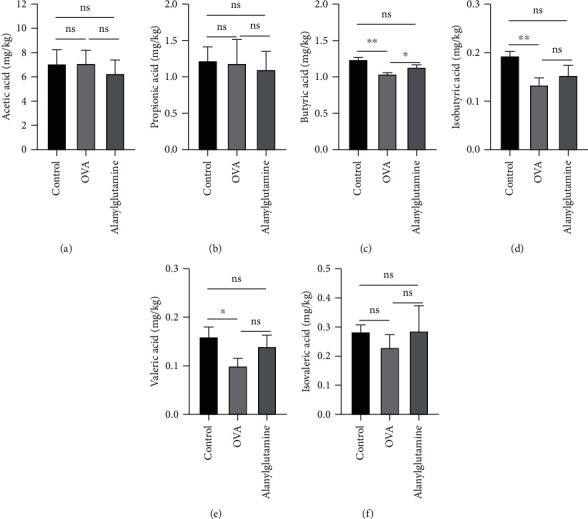
Effects of Alanylglutamine on bacterial metabolites in the stool in OVA-induced allergic asthma mice: (a) acetic acid; (b) propionic acid; (c) butyric acid; (d) isobutyric acid; (e) valeric acid; (f) isovaleric acid. ^∗^*P* < 0.05; ^∗∗^*P* < 0.01; ns: not significant.

Bacterial metabolites such as acetic acid (Figure 4(a)), propanoic acid (Figure 4(b)), butyric acid (Figure 4(c)), isobutyric acid (Figure 4(d)), valeric acid (Figure 4(e)), and isovaleric acid (Figure 4(f)) in the stool were further analyzed. OVA treatment markedly reduced fecal butyric acid, isobutyric acid, and valeric acid concentrations (*P* < 0.05). However, dietary supplementation with Alanylglutamine enhanced the butyric acid level (*P* < 0.05), indicating that butyric acid may involve in alleviating the role of Alanylglutamine in OVA-induced allergic asthma mice.

### 3.4. Alanylglutamine Treatment Failed to Alleviate OVA-Induced Allergic Asthma in Antibiotic-Challenged Mice

**Figure 5:**
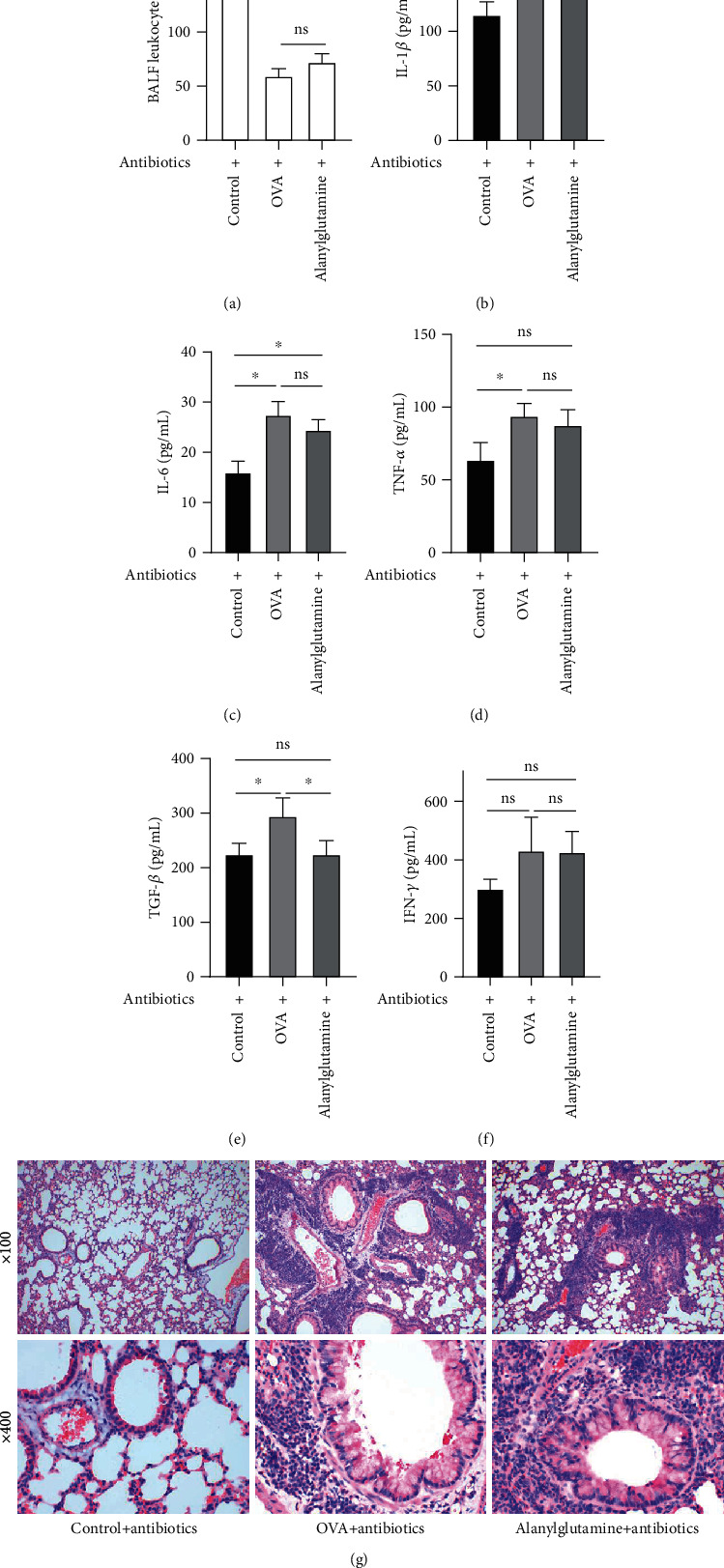
Alanylglutamine treatment failed to alleviate OVA-induced allergic asthma in antibiotic-challenged mice. The expression of inflammatory cytokines by ELISA in the groups of control+antibiotic, OVA+antibiotic, and Alanylglutamine+antibiotic mice: (a) BALF leukocyte count; (b) IL-1*β*; (c) IL-6; (d) TNF-*α*; (e) TGF-*β*; (f) IFN-*γ* levels from BALF; (g) lung tissue morphology study measured by H&E staining. *n* = 8. ^∗^*P* < 0.05; ^∗∗^*P* < 0.01; ns: not significant.

To further investigate the association of Alanylglutamine with gut microbiota in asthma, antibiotics were employed to eliminate the microbiota. Similarly, the leukocyte in BALF was markedly reduced (Figure 5(a)). The concentrations of IL-1*β*, IL-6, TNF-*α*, and TGF-*β* were elevated in OVA and antibiotic-cotreated mice (Figures 5(b)–5(f)). However, Alanylglutamine failed to affect leukocyte, IL-1*β*, IL-6, and TNF-*α* level in BALF, but it alleviated TGF-*β* concentration in antibiotic-challenged mice. H&E staining also showed no obvious alleviating effect on the inflammatory infiltration in Alanylglutamine and antibiotic cotreatment (Figure 5(g)). In summary, these results indicated gut microbiota might involve in the role of Alanylglutamine in the OVA-induced allergic asthma.

**Figure 6:**
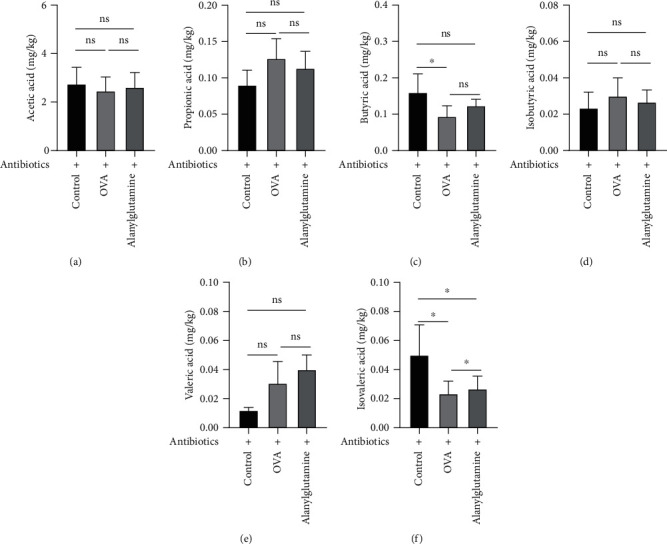
Amino acid content (mg/kg) in the groups of control+antibiotic, OVA+antibiotic, and Alanylglutamine+antibiotic mice: (a) acetic acid; (b) propionic acid; (c) butyric acid; (d) isobutyric acid; (e) valeric acid; (f) isovaleric acid. ^∗^*P* < 0.05; ns: not significant.

SCFA content analysis in the stool was further conducted to investigate the effects of Alanylglutamine treatment in asthma mice with antibiotics (Figures 6(a)–6(f)). The levels of butyric acid and isovaleric acid in asthma mice were significantly downregulated (Figures 6(c) and 6(f)). It was verified that butyric acid may have important effects on the regulation procedure of Alanylglutamine or intestinal microorganism. Therefore, in the following experiments, butyric acid was directly added to the mice to examine its exact functional mechanism.

### 3.5. Effects of Butyric Acid in OVA-Induced Allergic Asthma Mice

**Figure 7:**
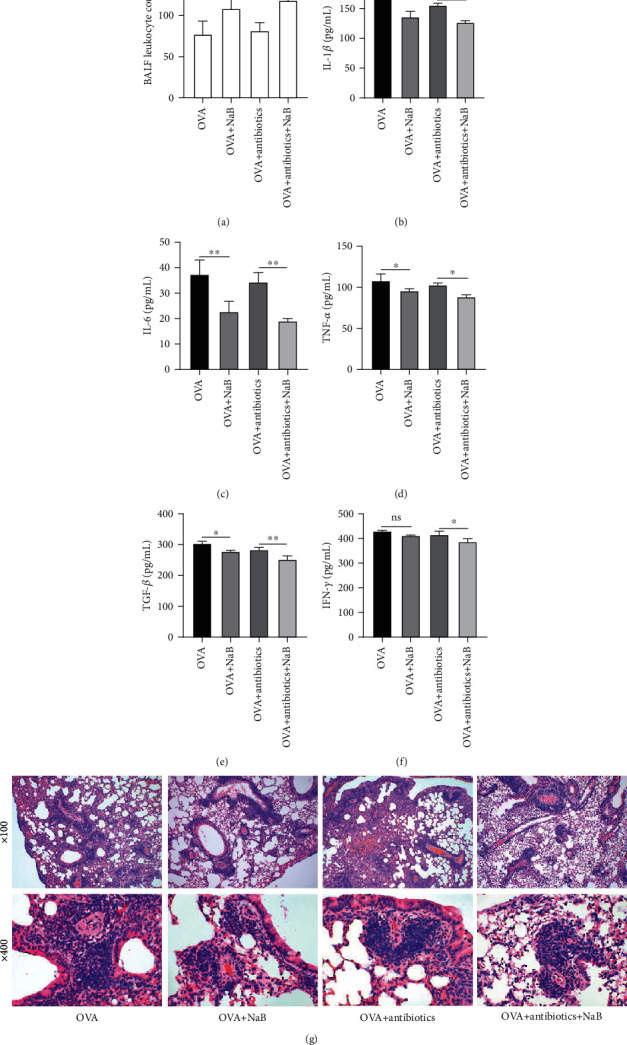
Effects of butyric acid on OVA-induced allergic asthma through cytokine productions in BALF and inflammatory infiltration in the lung tissue. The expression of inflammatory cytokines by ELISA in the groups of OVA, OVA+NaB, OVA+antibiotic, and OVA+antibiotic+NaB mice: (a) BALF leukocyte count; (b) IL-1*β*; (c) IL-6; (d) TNF-*α*; (e) TGF-*β*; (f) IFN-*γ* level from BALF; (g) lung tissue morphology study measured by H&E staining. *n* = 8. ^∗^*P* < 0.05; ^∗∗^*P* < 0.01; ns: not significant.

Microbial and metabolite analyses indicated that gut microbiota and butyric acid might involve in the role of Alanylglutamine in OVA-induced allergic asthma mice. Thus, NaB was further administrated to identify the potential mechanism. An OVA-induced allergic asthma model was induced with or without antibiotics. BALF leukocyte and cytokines were further tested (Figures 7(a)–7(f)). The results showed that NaB treatment significantly enhanced BALF leukocyte contents. The OVA+antibiotic+NaB group also enhanced BALF leukocyte content. It reduced the IL-1*β*, IL-6, TNF-*α*, and TGF-*β* level in OVA-induced allergic asthma both in antibiotic-free and antibiotic-treated mice notably, indicating an anti-inflammatory effect in the OVA-induced allergic asthma model. Compared with the model group, NaB treatment showed reduced inflammatory infiltration on the H&E-stained sections of lung tissue (Figure 7(g)), supporting the effect of butyric acid on leukocyte count. The above results indicated that both NaB and antibiotics had a certain anti-inflammatory effect. The anti-inflammatory treatment of NaB was more effective than that of antibiotics, and their combination had the most effective treatment.

**Figure 8:**
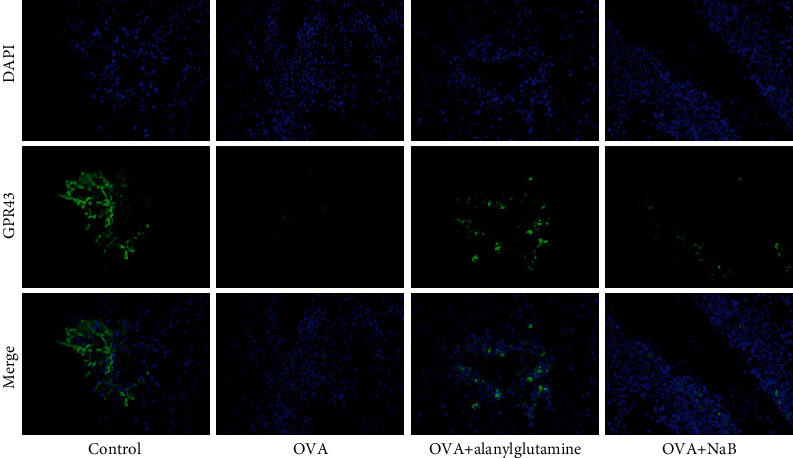
Immunofluorescence staining for the expressions of GPR43, DAPI, and their merge results in the groups of control, OVA, OVA+Alanylglutamine, and OVA+NaB mice. *n* = 8.

Considering that GPR43 served as the specific receptor of butyric acid, lung tissues were collected for analysis of GPR43 expression using the immunofluorescence (Figure 8). In the asthma model (OVA), the GPR43 expression was reduced compared with the control group, while the treatment of OVA+Alanylglutamine and OVA+NaB markedly enhanced the expression of GPR43 (Figure 8). The immunofluorescence staining results further confirmed the protective role of Alanylglutamine in asthma mice, which might be associated with the gut microbiota-butyric acid-GPR43 pathway.

### 3.6. Alanylglutamine Inhibited the NF-*κ*B Pathway and STAT3 Pathway

**Figure 9:**
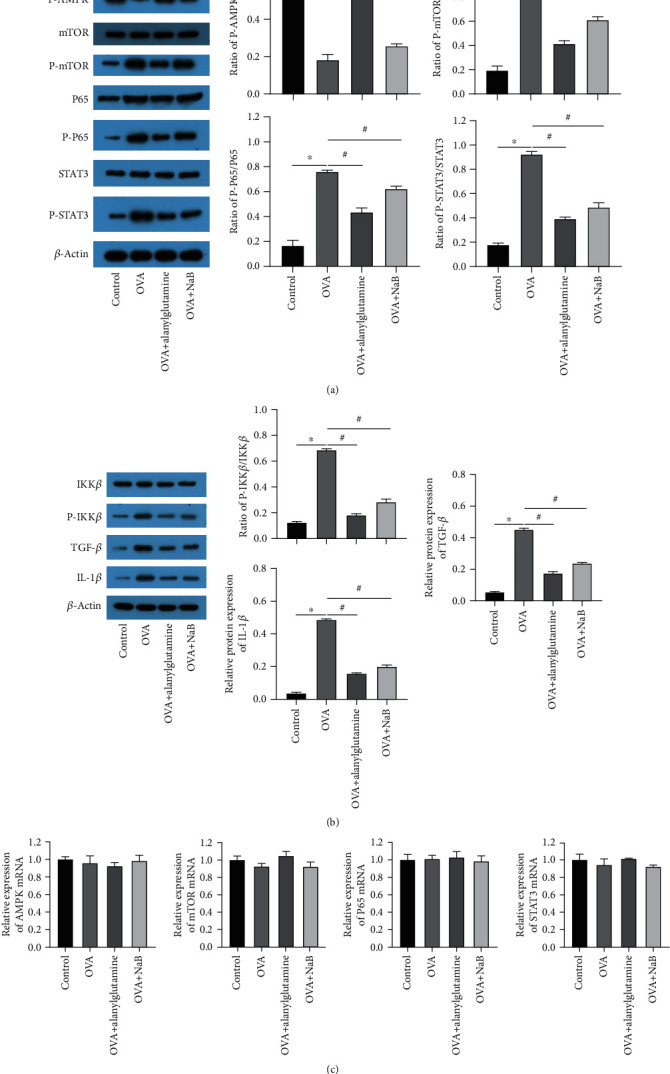
Alanylglutamine inhibited the NF-*Κ*B pathway and STAT3 pathway. (a) The expressions of AMPK, P-AMPK, mTOR, P-mTOR, P65, P-P65, STAT3, P-STAT3, and *β*-Actin and quantitative ratio statistics of P-AMPK/AMPK, P-mTOR/mTOR, P-P65/P65, and P-STAT3/STAT3. (b) The expressions of IKK*β*, P-IKK*β*, TGF-*β*, and IL-1*β* and their corresponding quantitative statistics. (c) Related mRNA expressions of AMPK, mTOR, P65, and STAT3 by RT-qPCR in the groups. *n* = 3. ^∗^*P* < 0.05 vs. the control; ^#^*P* < 0.05 vs. the OVA group.

To clarify the molecular mechanisms of Alanylglutamine in OVA-induced allergic asthma, we further collected the lung samples from control, OVA, Alanylglutamine-treated asthma mouse (OVA+Alanylglutamine), and NaB treatment groups (OVA+NaB). We analyzed the protein and mRNA expressions of several relative signaling pathways through Western blot (Figures 9(a) and 9(b)) and RT-qPCR (Figure 9(c)). It was shown that both Alanylglutamine and butyric acid could affect AMPK, NF-*κ*B, mTOR, and STAT3 signaling pathways. In contrast to the OVA group, Alanylglutamine activated the protein expressions of P-AMPK/AMPK. It inhibited the protein expressions of P-mTOR/mTOR, P-P65/P65, and P-STAT3/STAT3, with similar effects from butyric acid (Figure 9(a)). Abnormal activation of NF-*κ*B is usually closely related to IKK*β* phosphorylation. Therefore, the upstream and downstream pathway proteins of NF-*κ*B were tested by Western blot. Compared with the OVA group, Alanylglutamine suppressed the protein expressions of P-IKK*β*/IKK*β*, TGF-*β*, and IL-1*β* (Figure 9(b)). The results of RT-qPCR indicated that Alanylglutamine failed to affect the mRNA expression of AMPK, mTOR, P65, and STAT3 (Figure 9(c)). In summary, the results demonstrated that Alanylglutamine activated the AMPK pathway and inactivated the mTOR, P65, and STAT3 pathway.

## 4. Discussion

Colonized gut microbiota is presently recognized as potential mediators of host immune responses in different diseases. The remodeling of microbiota contributed to different effects on inflammation in the lung. For instance, it was confirmed that gut microbiota was overtly changed. Susceptibility to the TH2 model of allergic asthma was also increased, with perinatal exposure to vancomycin, but not streptomycin [16]. The importance of immune regulation by commensal microbiota in the respiratory mucosa via inflammasome activation was also revealed. Alanylglutamine was reported to reduce muscle wastage of alanine and glutamine in anaesthetized dogs after operation [17]. In oxidatively stressed Caco-2 cells, the dipeptide could hold PepT1-mediated transport [18]. Based on the relationship between gut microbiota and immunity, we analyzed the roles of the dipeptide Alanylglutamine in OVA-induced asthma with allergic lung inflammation. Administrations of Alanylglutamine with different feeding routes by parenteral or enteral led to an enhanced plasma glutamine response compared to baseline [19]. Herein, we discovered that the uptake of Alanylglutamine changed the gut microbiota, especially in the OVA-induced asthma model.

Probiotics are used to modify the intestinal flora, which is beneficial for improving the nonalcoholic fatty liver disease (NAFLD) in ob/ob mice [20]. NAFLD is characterized by intestinal bacterial overgrowth. Thus, we postulated that Alanylglutamine-mediated changes of probiotics might improve the inflammation condition through intestinal flora modification. Indeed reshaping of the gut microbiota as well as increased levels of short-chain fatty acids by high-fiber feeding exhibited beneficial roles in food allergy with increased Treg differentiation [21]. SCFA are microbial metabolites derived from bacterial fermentation serving as a sign of gut health, which are also considered to modulate chronic inflammation illnesses. SCFA-producing bacteria are the connection between microbiota functions and epigenetic regulation of inflammatory mechanisms [22]. SCFAs, such as acetate, propionate, and butyrate, are produced when dietary fiber is fermented by gut microbiota. As for butyrate, it was reported that sodium butyrate is capable of inducing apoptosis in tumor cell lines [23]. Recent studies have unveiled that sodium butyrate and other short-chain fatty acids can guard against inflammation in colon diseases. Butyrate in CNS is anti-inflammatory in brain-derived microglial cells. However, it shows proinflammation in the microglial cell line, which may be related to the anticancer properties of butyrate observed in tumor cells [24]. In this study, we also found that Alanylglutamine mediated SCFA during remodeling of gut microbiota and protected against airway allergy such as asthma.

In the respiratory tract, there was synergistic antitumor activity against lung cancer cells with a combination therapy of lovastatin and butyrate in vitro [25]. LPS-induced acute lung injury was also ameliorated by sodium butyrate with reduced HMGB1 release [26–28]. In this study, we found that the butyrate displayed protective effects on asthma. After the intervention of the chemicals, the signaling pathways including AMPK, mTOR, P65, and STAT3 were also affected. It was noticed that Alanylglutamine had almost the same effects on asthma, compared with butyrate. The AMPK phosphorylation was upregulated markedly, while NF-*κ*B, STAT3, and Akt/mTOR signals were downregulated significantly after the Alanylglutamine treatment. Alanylglutamine also inhibited the phosphorylation of IKK*β* and the protein expression of TGF-*β* and IL-1*β*. The effects on molecular signaling pathways of AMPK, mTOR, P65, and STAT3 are also consistent with the previous researches, in which Plumbagin protects liver against fulminant hepatic failure and chronic liver fibrosis in LX-2 cells [29].

Although we performed analysis in several signaling pathways, the exact molecular pathways involved in the pathogenesis and treatment of various diseases are quite complex and complicated. In a previous study, the scientist demonstrated that diet supplemented with baicalin could alleviate oxidative stress and enhance nutrition absorption in deoxynivalenol-challenged pigs. The effects of baicalin might be related to the inhibition of NF-*κ*B and activation of the mTOR pathway, which are different from our results [30–33]. Another study showed that anti-inflammatory and antioxidative stress effects of baicalin about atherosclerosis might be in connection with inhibiting the NF-*κ*B and p38 MAPK signaling pathways [33]. Considering the complexities in the signaling pathways involved in the functional procedures from Alanylglutamine, we plan to further study the relevant molecular mechanisms in its therapy to asthma in the future research.

It is established that intestinal flora closely involves in the development of asthma [34], which is often accompanied by inflammatory reaction. In the peripheral blood of children with asthma, the possibility of intestinal flora disorders and gastrointestinal discomfort symptoms increases, with the elevation in the levels of inflammatory factors such as TNF-*α* and IL-6 [35]. In a previous study, Stephanie et al. showed that TNF-*α*, IL-33, and IL-13 had a crucial role in treatment of asthma in obese mice, of which the gut microbiome was altered in contrast to the control mice [36]. In this study, we found that Alanylglutamine could reverse the changes of intestinal flora and inflammatory factors, inducing by OVE. Due to funding and time limitations, we did not provide the molecular evidence for the direct correlation between gut microbiota and the symptom. In the future work, we will perform in-depth analysis on this aspect and hope to further enhance the treatment effects of asthma with related drugs.

## 5. Conclusions

We established the relationship among Alanylglutamine dipeptide, SCFA, and antibiotics in the asthma model through a series of experimental verifications. Our results implicated that Alanylglutamine might be beneficial for asthma, and its effect was achieved through regulation on microbiota and the derived metabolites of butyric acid. Moreover, the therapeutic effects might involve in the regulation of AMPK, NF-*κ*B, mTOR, and STAT3 signaling pathways. These findings will help identify the effective therapeutic direction to alleviate allergic inflammation of the lungs and airways.

## Data Availability

The authors confirm that all data underlying the findings are available. All relevant data are within the paper or its supplementary materials.

## Supplementary Materials

Supplementary Materials Supplementary Figure 1: microbiota at the genus level (top 20) was analyzed, including Corynebacterium, Adlercreutzia, Bacteroides, Parabacteroides, Prevotella, Odoribacter, Prevotella, Sporosarcina, Staphylococcus, Lactobacillus, Streptococcus, Turicibacter, Dehalobacterium, Coprococcus, Dorea, Ruminococcus, Oscillospira, Butyricimonas, Allobaculum, and Sutterella. 11 genera were markedly altered in response to OVA or Alanylglutamine treatment. ^∗∗^*P* < 0.01; ^∗∗∗^*P* < 0.001; ns: not significant.
