# Supplementary figures and images for: Alanylglutamine Relieved Asthma Symptoms by Regulating Gut Microbiota and the Derived Metabolites in Mice
Source: Oxid Med Cell Longev. 2020 Dec 29;2020:7101407. doi: 10.1155/2020/7101407 (PMC7785351; doi:10.1155/2020/7101407)

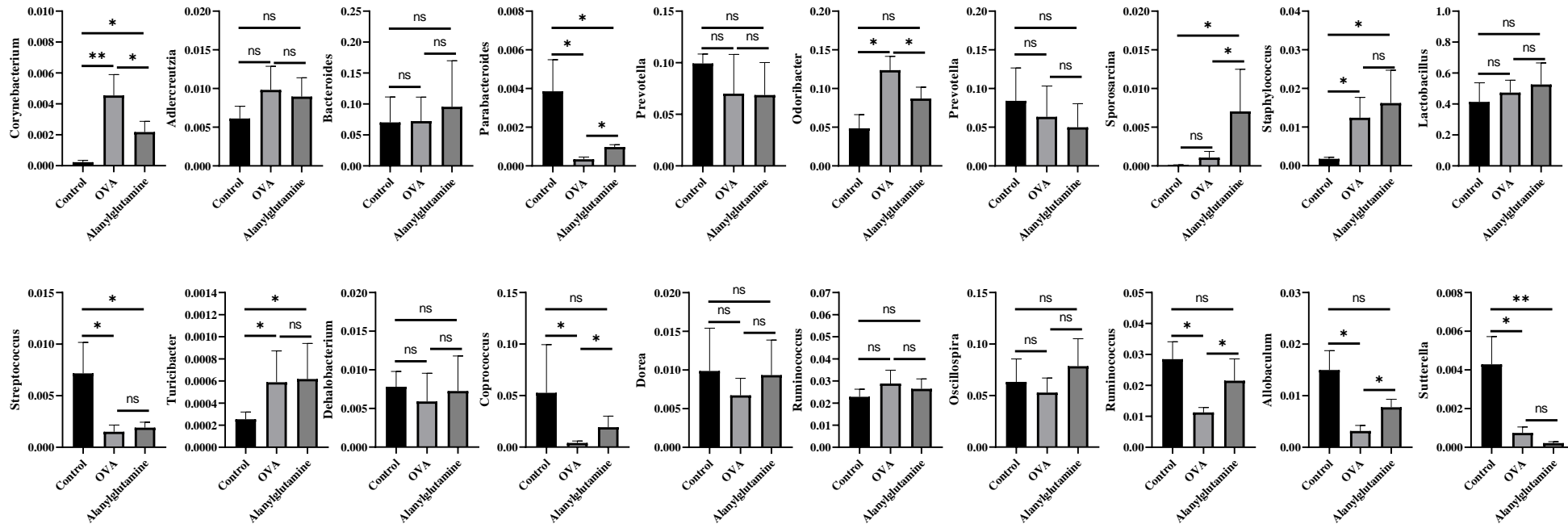

Supplement: Supplementary Materials — Supplementary Figure 1: microbiota at the genus level (top 20) was analyzed, including Corynebacterium, Adlercreutzia, Bacteroides, Parabacteroides, Prevotella, Odoribacter, Prevotella, Sporosarcina, Staphylococcus, Lactobacillus, Streptococcus, Turicibacter, Dehalobacterium, Coprococcus, Dorea, Ruminococcus, Oscillospira, Butyricimonas, Allobaculum, and Sutterella. 11 genera were markedly altered in response to OVA or Alanylglutamine treatment. ∗∗P < 0.01; ∗∗∗P < 0.001; ns: not significant. [file 7101407.f1.pdf]
